# Effect of Nighttime Exercise on Sleep Quality Among the General Population in Riyadh, Saudi Arabia: A Cross-Sectional Study

**DOI:** 10.7759/cureus.41638

**Published:** 2023-07-10

**Authors:** Eid H Alkhaldi, Safar Battar, Sulaiman I Alsuwailem, Khalid S Almutairi, Waleed K Alshamari, Ahmed H Alkhaldi

**Affiliations:** 1 Preventive Medicine, Ministry of Health, Riyadh, SAU; 2 Preventive Medicine, King Khalid University Medical City, Abha, SAU; 3 Preventive Medicine, Saudi Public Health Authority, Riyadh, SAU; 4 Preventive Medicine, Jouf University-College of Medicine, Al-Jouf, SAU

**Keywords:** saudi arabia, physical activities, the effect of physical exercises on sleep quality, sleep quality, nighttime exercises

## Abstract

Introduction

Engaging in physical activity has been proven to have health benefits, with a positive impact on sleep quality. While the timing of exercise plays a significant role in determining its effect on sleep, nighttime exercise still needs to be explored, especially in Saudi Arabia. This study assessed the effect of nighttime exercise on sleep quality among the general population in Riyadh, Saudi Arabia.

Methods

A cross-sectional study was conducted on people performing physical exercises in the gym, training, and walking places using a self-administered questionnaire evaluating exercising behaviors and sleep quality. Comparisons were performed using the Chi-square test and ANOVA, and p<0.05 was considered for significance.

Results

We enrolled 385 participants, among whom 53.8% were male, and 47.2% were female. The mean age was 28.2±7.85 years, and mostly aged 25-29 years (24.7%), followed by 20-24 years old (21.3%). Most had university degrees (61.3%) and were also employed (60%). Of all participants, the majority were overweight and obese (61.3%). Most (n=225) participants practiced vigorous physical exercise, and the mean Pittsburgh Sleep Quality Index (PSQI) global score was 7.37±3.49 points. Evening vigorous (r= 0.25, p=0.038) and moderate (r=0.30, p=0.025) physical exercise sessions lasting > 90 min had a significant positive correlation with poor sleep quality (high PSQL score). There was no statistically significant correlation between sleep quality and other variables (p>0.05).

Conclusion

This study found that participants had poor sleep quality, and lengthy, intense evening exercises had a negative effect on sleep. Incorporating regular exercise tailored to individual preferences and encouraging people to widen the time interval between exercise and bedtime could improve sleep quality.

## Introduction

Studies conducted over the last decade have shown that poor sleep quality is associated with an increased risk of serious medical conditions such as cardiovascular disease and cancer and an increased likelihood of developing depression [1]. Despite its widespread usage, the word "sleep quality" has not been precisely defined in the field of sleep medicine. Key determinants (sleep latency, frequency of awakenings >5 minutes, wake following sleep initiation, and sleep efficiency) of quality sleep in healthy persons were reported by the National Sleep Foundation (NSF) without considering sleep architecture or nap-related factors [2]. Physical activity and sleep are interdependently associated via many psychological and physiological mechanisms [3,4]. Acute exercise of low to moderate intensity has been demonstrated to improve sleep amount in a variety of studies [5]. Late-night high-intensity exercises (HIE) have been demonstrated to enhance psychophysiological stress [6], alter circadian phase [7], heighten arousals [8], trigger sympathetic hyperactivity, raise body temperature, and/or disrupt the normal nocturnal drop in core body temperature [9], which might disrupt sleep quality. Other studies have documented modest exercise-induced muscle damage, subjective muscular soreness, and a high evaluation of tiredness after acute nighttime HIE compared to no-exercise settings. Thus, the persistence of post-exercise psychophysiological reactions that continue to bedtime could indicate sleep start disturbance [10]. On the other hand, exercise in the evening may enhance sleep via anxiety reduction and antidepressant benefits [11,12].

The positive impact of exercise on sleep has been the subject of several studies, but the connection between exercise intensity in the evening and restful sleep has not been specifically studied [11]. Even though it is still debatable, a number of aspects of physical activity have been reported to enhance sleep, including an increase in body temperature before tonight, modifications in cortisol and growth hormone release, and enhanced mood [5,12]. According to our search results, fewer studies were conducted regarding the same subjects, while a sedentary lifestyle in Saudi Arabia is associated with poor exercise, sleep, and quality of life [13]. Campaigns have been conducted, inciting Saudis to exercise more [14-16]. Therefore, this study evaluated the effect of nighttime exercise on sleep quality among the general population in Riyadh, Saudi Arabia, to inform measures to ensure the population exercises with minimal sleep problems.

## Materials and methods

Study design

This was an analytical cross-sectional study based on a self-administrated questionnaire, and it was conducted in Riyadh, Saudi Arabia, from January 2023 to May 2023 at gyms and general training places distributed in various parts of Riyadh City.

Participants

The study targeted all people aged 20-50 from different nationalities who were attendees of gyms, walking, and training places. The workers of the gym, walking, and training institutions were excluded. Moreover, those below or above the age range were excluded since they are mostly not found in the settings of our study.

Sample size and recruitment

The sample size was calculated using the Raosoft calculator, considering the proportions of questionnaire accuracy as 50% and a margin of error of 5%, with a confidence interval of 95%, which gave 385 participants, as the minimum sample size.

We used a stratified sampling technique to select eligible participants. Riyadh City was divided into five regions (central, southern, eastern, western, and northern regions). Then, two gyms, two general training, and two walking places were randomly chosen from each region. Subsequently, 13 participants were selected from each gym, general training, and walking place using a simple random sampling technique, making 390 participants in total.

Data collection procedures and tool

A structured questionnaire was used to collect the data from the included participants and was based on previous studies by Buman et al. [17], and Buysse et al. [18]. The questionnaire had three parts. The first assessed socio-demographic factors, including age, nationality, employment status, education level, marital status, and body mass index. The second part assessed exercising behaviors using an adapted International Physical Activity Questionnaire to categorize physical activity as either vigorous, moderate, or light [19]. Each participant was permitted to endorse numerous activities across several intensity categories by responding independently to each question regarding exercise intensity. After each intensity question, participants were asked when they typically engaged in that activity and responded by selecting: (a) more than eight hours before bedtime ("morning"); (b) between four and eight hours before sleep (afternoon); or (c) less than four hours before bedtime ("evening"). Each person was assigned to one of three categories based on their preference for morning, afternoon, or evening exercise: intense, moderate, or mild. The third part included questions regarding sleep quality within the previous two weeks. One 4-item Likert-type question with 'very good,' 'pretty good,' 'fairly terrible,' and 'very awful' answer possibilities was used to assess general sleep quality using The Pittsburgh Sleep Quality Index (PSQI) [18].

The questionnaire was translated into Arabic from English and reviewed by experts. It was piloted on 30 participants with similar characteristics as the main sample, and the results were used for word clarity and improvement only. The Cronbach's alpha reliability test of the questionnaire showed a score of 0.81. The investigators visited the gyms, walking, and training places, and after explaining to the participants, they gave them hard copies of the questionnaire to fill out for themselves. 

Statistical analysis

The statistical analysis was done using the Statistical Package for Social Sciences version 22 (SPSS Inc., Chicago, IL, USA). Comparison between different groups regarding categorical variables was tested using the Chi-square test. Quantitative data were described using mean and standard deviation for normally distributed data. For normally distributed data, comparisons between more than two populations were analyzed, and F-test (ANOVA) was used. The significance of the obtained results was judged at the 5% level. The correlation coefficient (r) test was used to find the association between sleep quality and physical exercises.

Ethical approval

Written approval was provided by the King Fahad Medical City Institutional Review Board (IRB) with reference number: 23-039E. 

## Results

This study got responses from 385 participants, of whom 53.8% were male and 46.2% were female. The majority of the participants were aged 25-29 years (24.7%), and the mean age was 28.2±7.85 years. The majority of participants had a university degree (61.3%) and were employed (60%). The anthropometric measurements showed that normal weight, overweight, and obesity represented 34.8%, 38.2%, and 23.1% of the population, respectively (Table 1).

**Table 1:** Sociodemographic characteristics of participants *Mean±S.D: Mean*±S*tandard Deviation; BMI: Body mass index*

Sex	Number	%
Male	207	53.8
Female	178	46.2
Age		
20-24	82	21.3
25-29	95	24.7
30-34	75	19.5
35-39	40	10.4
40-44	51	13.2
45-50	42	10.9
Range (Mean±S.D)	20-50 28.2±7.85	
Education		
Secondary	70	18.2
University	237	61.6
High	78	20.3
Occupation		
Student	71	18.4
Employee	233	60.5
Free work	22	5.7
Not working	50	13.0
Retired	9	2.3
Marital status		
Single	185	48.1
Married	185	48.1
Divorced	12	3.1
Widow	3	0.8
BMI category		
Underweight	15	3.9
Normal weight	134	34.8
Overweight	147	38.2
Obese	89	23.1
Total	385	100.0

We found that most (n=225) participants practiced vigorous intense physical exercise, 62 practiced moderate physical exercise, and 98 practiced light physical exercise. Overall, most participants exercised in the evening (n=204). Of those exercising in the morning, and afternoon, most exercised for 30-59 minutes (n=35 and n=43, respectively), while in the evening, most exercised for less than 30 minutes (n=97) (Table 2).

**Table 2:** The intensity, duration, and time of physical exercises practiced by participants n: Frequency/Number of participants; %: Percentage; No: Number of respondents

Time of physical exercise	Duration of physical exercise	Type of physical exercise						Total	
		Vigorous “n=225”		Moderate “n=62”		Light “n=98”			
		No	%	No	%	No	%	No	%
Morning	< 30 min	8	19.5	5	35.7	16	64.0	29	36.3
	30-59 min	21	51.2	7	50.0	7	28.0	35	43.8
	60-90 min	7	17.1	2	14.3	1	4.0	10	12.5
	> 90 min	5	12.2	0	0.0	1	4.0	6	7.5
	Total	41	100.0	14	100.0	25	100.0	80	100.0
Afternoon	< 30 min	21	31.8	6	31.6	10	62.5	37	36.6
	30-59 min	27	40.9	11	57.9	5	31	43	24.6
	60-90 min	12	18.2	2	10.5	0	0.0	14	13.9
	> 90 min	6	9.1	0	0.0	1	6.3	7	6.9
	Total	66	100.0	19	100.0	16	100.0	101	100.0
Evening	< 30 min	45	38.1	14	38.3	38	66.7	97	47.5
	30-59 min	40	33.9	13	44.8	13	22.8	66	32.4
	60-90 min	21	17.8	2	6.9	2	3.5	25	12.3
	> 90 min	12	10.2	0	0.0	4	7.0	16	7.8
	Total	118	100.0	29	100.0	57	100.0	204	100.0

The mean PSQI global score was 7.37±3.49 points, with 67.3% of participants scoring over 5.0 points (indicating a poor sleeper) (Table 3). 

**Table 3:** Pittsburgh Sleep Quality Index scores among participants n (%): Frequency (Percentage); SD: Standard Deviation; PSQI: Pittsburgh Sleep Quality Index

	Range	Mean±S.D.
Sleep duration	0.0-3.0	1.43±1.06
Sleep disturbance	0.0-3.0	1.12±0.63
Sleep latency	0.0-3.0	1.58±1.00
Daytime dysfunction due to sleepiness	0.0-3.0	0.49±0.76
Sleep efficiency	0.0-3.0	1.43±1.06
Overall sleep quality	0.0-3.0	1.01±0.74
Sleep medication use	0.0-3.0	0.30±0.73
Global PSQI score	0.0-20.0	7.37±3.49
PSQI category	n (%)	
Good	126 (32.7%)	
Poor	259 (67.3%)	

There was no statistically significant correlation between sleep quality and socio-demographic and anthropometric characteristics (p > 0.05) (Table 4).

**Table 4:** Correlation between sleep quality and basic characteristics of participants No: Number of respondents; %: Percentage; X^2^: Chi-square test value

	Sleep Quality				Total		X^2^	P value
	Good		Poor					
	No	%	No	%	No	%		
Sex							0.859	0.207
Male	72	57.1	135	52.1	207	53.8		
Female	54	42.9	124	47.9	178	46.2		
Age							3.840	0.573
20-24	22	17.5	60	23.2	82	21.3		
25-29	37	29.4	58	22.4	95	24.7		
30-34	22	17.5	53	20.5	75	19.5		
35-39	15	11.9	25	9.7	40	10.4		
40-44	17	13.5	34	13.1	51	13.2		
45-50	13	10.3	29	11.2	42	10.9		
Education							0.174	0.917
Secondary	23	18.3	47	18.1	70	18.2		
University	76	60.3	161	62.2	237	61.6		
High	27	21.4	51	19.7	78	20.3		
Occupation							2.735	0.603
Student	18	14.3	53	20.5	71	18.4		
Employee	81	64.3	152	58.7	16	60.5		
Free work	6	4.8	16	6.2	22	5.7		
Not working	18	14.3	32	12.4	50	13.0		
Retired	3	2.4	6	2.3	9	2.3		
Marital status							2.137	0.545
Single	59	46.8	126	48.6	185	48.1		
Married	64	50.8	121	46.7	185	48.1		
Divorced	3	2.4	9	3.5	12	3.1		
Widow	0	0.0	3	1.2	3	0.8		
BMI category							4.278	0.233
Underweight	4	3.2	11	4.2	15	3.9		
Normal weight	37	29.4	97	37.5	134	34.8		
Overweight	49	38.9	98	37.8	147	38.2		
Obese	36	28.6	53	20.5	89	23.1		

There was no statistically significant correlation between PSQI scores and physical exercise intensity and time (p<0.05) (Table 5).

**Table 5:** Correlation between different items of the Pittsburgh Sleep Quality Index (PSQI) mean scores and physical exercise intensity and time *PSQI: Pittsburgh Sleep Quality Index; ANOVA: Analysis of Variance;* *SD: Standard Deviation*

Physical exercise intensity	Time of physical exercise	Sleep duration (Mean±SD)	Sleep disturbance (Mean±SD)	Sleep latency (Mean±SD)	Daytime dysfunction due to sleepiness (Mean±SD)	Sleep efficiency (Mean±SD)	Overall sleep quality (Mean±SD)	Sleep medication use (Mean±SD)	Global PSQI score (Mean±SD)
Vigorous	Morning	1.52±1.12	1.13±0.61	1.54±0.96	0.53±0.82	1.52±1.12	1.08±0.79	0.44±0.90	7.76±3.49
	Afternoon	1.42±1.04	1.27±0.65	1.64±1.00	0.53±0.75	1.42±1.04	1.03±0.80	0.33±0.75	7.65±3.63
	Evening	1.29±1.05	1.07±0.65	1.32±1.13	0.46±0.64	1.29±1.05	0.66±0.69	0.17±0.54	6.27±3.52
Moderate	Morning	1.76±0.95	1.03±0.57	1.76±0.95	0.55±0.78	1.76±0.95	1.07±0.53	0.31±0.76	8.24±3.30
	Afternoon	1.58±1.07	1.05±0.62	1.68±1.00	0.47±0.77	1.58±1.07	1.00±0.67	0.05±0.23	7.42±3.31
	Evening	1.29±0.91	1.07±0.47	1.29±0.83	0.36±0.63	1.29±0.91	0.93±0.27	0.14±0.36	6.36±2.79
Light	Morning	1.52±1.12	1.12±0.78	2.00±0.87	0.36±0.70	1.52±1.12	1.16±0.75	0.36±0.76	8.04±3.55
	Afternoon	1.56±0.96	1.13±0.72	1.31±1.20	0.75±0.93	1.56±0.96	1.31±0.87	0.19±0.40	7.81±3.15
	Evening	1.12±1.00	1.02±0.61	1.63±1.05	0.39±0.70	1.12±1.00	0.93±0.68	0.18±0.60	6.39±3.48
ANOVA		1.6	1.283	0.787	0.397	1.6	0.225	1.952	0.601
P value		0.203	0.278	0.456	0.672	0.203	0.798	0.143	0.549

Table 6 shows the correlation between the duration of physical exercise and global PSQL score in terms of intensity and time. It was found that evening vigorous (p=0.038) and moderate physical exercise sessions (p=0.025) lasting > 90 min had a significant positive correlation with PSQI score, which means the long-lasting physical exercise in the evening, if vigorous or moderate, negatively affected sleep quality. However, there was no significant correlation between light physical exercise intensity and PSQI score.

**Table 6:** Correlation between duration of physical exercise and global PSQI score in terms of intensity and time *Statistically significant

Physical exercise intensity	Time of physical exercise	Correlation coefficient	P value
Vigorous	Morning	0.027	0.591
	Afternoon	0.108	0.211
	Evening	0.254	0.038*
Moderate	Morning	0.113	0.205
	Afternoon	0.171	0.169
	Evening	0.301	0.025*
Light	Morning	0.08	0.311
	Afternoon	0.017	0.684.
	Evening	0.099	0.711

## Discussion

Sleep is vital to overall health and well-being and plays a crucial role in the body's physical and mental restoration, cognition, and immune function [20]. Many studies demonstrate that poor sleep increases the chance of developing multiple illnesses [8,21], eventually shortening life expectancy [22]. There has been a growing interest in understanding the relationship between exercise and sleep quality [23]. Rapid urbanization, lifestyle changes, and increased exposure to electronic devices have contributed to sleep disturbances among the population. According to a study conducted in Saudi Arabia, approximately 30-40% of adults experience poor sleep quality, adversely affecting their daily lives and overall well-being [13,24]. Hence, exploring factors influencing sleep quality is of utmost importance. Our study explored the impact of nighttime exercise on sleep quality among the general population of Saudi Arabia.

We found that almost two-thirds of the participants performed vigorous physical exercises, which may be attributed to the type of audience, including those who go to the gym and other training and exercise places. Physical activity is an effective and affordable strategy for improving sleep [5]. Exercise stimulates the release of endorphins, reduces stress, and promotes relaxation, all of which can contribute to better sleep.

Recent meta-analyses on the impact of physical activity on sleep have demonstrated that regular exercise enhances a multitude of sleep characteristics, including sleep-onset latency, total sleep time, and sleep efficiency [5,24]. However, the timing of exercise plays a significant role in determining its effect on sleep. Nighttime exercise, in particular, has been a subject of interest due to its potential to enhance sleep quality. Several studies have investigated the impact of nighttime exercise on sleep quality, and the results have been mixed. A randomized crossover study found that participants who engaged in moderate-intensity exercise in the evening reported improved sleep quality [25]. Another study showed that sleep-onset latency improves following late-night exercise, although other investigators showed no differences in sleep patterns following evening exercise compared to a no-exercise group [26,27]. We found that 67.3% of the participants scored over 5.0 points on the PSQI score, indicating a poor sleeper, which might align with another study that showed that late-night activities contribute to poor sleep [17]. Another study conducted at Jasan University also found that 63.9% of the participants had poor sleep [13].

Research has demonstrated that evening high-intensity exercise does not interfere with sleep [28,29], which aligns with our findings, showing a statistically insignificant correlation between sleep quality and the intensity and time of exercises (all p<0.05). However, it was suggested that early evening, high-intensity exercise might improve sleep quality [29]. In contrast, we found that long-lasting physical exercise, if vigorous or moderate and performed in the evening, negatively affected sleep quality. However, low-intensity physical exercise had no statistically significant effect. This indicates that exercising intensively too close to bedtime may disrupt sleep quality. High-intensity exercise 3 hours before bedtime has been cautioned due to probable overnight sleep disturbance [6,30]. Therefore, it is generally recommended to complete exercise at least 4 hours before sleeping [31].

Our study has some limitations related to its design for consideration. The cross-section design is limited in proving causality and is prone to selection bias. With cross-sectional designs, controlling confounding factors and studying rare outcomes or exposures may be difficult. Therefore, we recommend extensive longitudinal studies to identify causal correlations or long-term effects of exercises on sleep quality.

## Conclusions

This study showed that most participants exercised in the evening and had poor sleep quality (over 5 PSQI global score points). Moderately and vigorously intense exercises that lasted long in the evening were associated with poor sleep quality. As public health officials in Saudi Arabia encourage people to exercise more to prevent sedentary lifestyle-related morbidities and reduce non-communicable diseases, these findings highlight the need to educate people on the right time and intensity of exercise to improve sleep quality. This could involve incorporating regular exercise into one's routine to ensure the physical exercises don't interfere. Further research is recommended extensively evaluate long-term effects and optimal exercise prescription fitting different demographics.
